# Multicenter Evaluation of the Bruker MALDI Biotyper CA System for the Identification of Clinical Aerobic Gram-Negative Bacterial Isolates

**DOI:** 10.1371/journal.pone.0141350

**Published:** 2015-11-03

**Authors:** Matthew L. Faron, Blake W. Buchan, Josh Hyke, Neil Madisen, Jennifer L. Lillie, Paul A. Granato, Deborah A. Wilson, Gary W. Procop, Susan Novak-Weekley, Elizabeth Marlowe, Joven Cumpio, Christen Griego-Fullbright, Sandra Kindig, Karen Timm, Stephen Young, Nathan A. Ledeboer

**Affiliations:** 1 Medical College of Wisconsin, Milwaukee, WI, United States of America; 2 Wisconsin Diagnostic Laboratory, Milwaukee, WI, United States of America; 3 Laboratory Alliance of Central New York, Fayetteville, NY, United States of America; 4 SUNY Upstate Medical University, Syracuse, NY, United States of America; 5 Cleveland Clinic, Cleveland, OH, United States of America; 6 Kaiser Permanente SCPMG Regional Reference Laboratories, North Hollywood, CA, United States of America; 7 Tricore Reference Laboratories, Albuquerque NM, United States of America; 8 Department of Pathology UNMHSC, Albuquerque, NM, United States of America; Indian Institute of Science, INDIA

## Abstract

The prompt and accurate identification of bacterial pathogens is fundamental to patient health and outcome. Recent advances in matrix-assisted laser desorption/ionization time-of-flight mass spectrometry (MALDI-TOF MS) have revolutionized bacterial identification in the clinical laboratory, but uniform incorporation of this technology in the U.S. market has been delayed by a lack of FDA-cleared systems. In this study, we conducted a multicenter evaluation of the MALDI Biotyper CA (MBT-CA) System (Bruker Daltonics Inc, Billerica, MA) for the identification of aerobic gram-negative bacteria as part of a 510(k) submission to the FDA. A total of 2,263 aerobic gram negative bacterial isolates were tested representing 23 genera and 61 species. Isolates were collected from various clinical sources and results obtained from the MBT-CA System were compared to DNA sequencing and/or biochemical testing. Isolates that failed to report as a "high confidence species ID" [log(score) ≥2.00] were re-tested using an extraction method. The MBT-CA System identified 96.8% and 3.1% of isolates with either a "high confidence" or a "low confidence" [log(score) value between 1.70 and <2.00] species ID, respectively. Two isolates did not produce acceptable confidence scores after extraction. The MBT-CA System correctly identified 99.8% (2,258/2,263) to genus and 98.2% (2,222/2,263) to species level. These data demonstrate that the MBT-CA System provides accurate results for the identification of aerobic gram-negative bacteria.

## Introduction

The early and accurate identification of bacterial infections is crucial to patient care to help guide antimicrobial therapy. For decades, clinical laboratories have routinely identified pathogenic bacteria by using a combination of bacterial culture and biochemical tests. Traditional methods are reliable, however, results are often delayed by hours to days from collection, can fail to distinguish closely related species, and result in incorrect identifications more often than other newer diagnostic methods such as molecular testing [1]. Molecular assays allow for the rapid identification that can eliminate the need for bacterial subculture [2, 3]. However, molecular assays are only capable of identifying organisms targeted by the test, are more costly than culture, and have higher complexity compared to standard biochemical identification methods.

MALDI-TOF MS protocols were developed two decades ago to analyze whole bacterial cells, but the technology has only recently been used for routine identification [4, 5]. Routine identification by MALDI-TOF MS is performed by generating mass spectra from whole bacterial cells composed primarily of ribosomal proteins [6, 7]. These protein profile spectra are compared to a reference library comprised of a wide range of organisms resulting in accurate species specific bacterial identification [7–9]. Overall, MALDI-TOF MS demonstrates equivalence to current identification methods and benefits from reduced cost per identification and reduced turnaround time [1, 10]. In 2008, the first commercially-available MALDI-TOF systems were available for research purposes; however, the lack of FDA-cleared systems has delayed incorporation into routine clinical microbiology laboratory practice in the United States [11–13].

In this study, we conducted a multicenter evaluation of the ability of the MALDI Biotyper CA (MBT-CA) System (Bruker Daltonics Inc, Billerica, MA) to identify clinically relevant gram negative, aerobic bacteria. The goal of this study was to provide evidence of substantial equivalence to devices on the market for the 510(k) submission of the MALDI Biotyper CA System using the manufacturer provided reference database. A total of 2,263 gram-negative aerobic bacteria collected from multiple sources were tested on the Bruker MBT-CA System. All test results were compared to a bi-directional sequencing method partially supplemented by biochemical testing. Bacterial isolates tested in this study comprised 23 different genera and 61 different species.

## Materials and Methods

### Collection of study specimens

Gram-negative bacteria recovered from any clinical specimen (i.e. urine, blood, etc.) were enrolled into the study after all routine laboratory procedures were performed. Enrolled organisms were isolated onto either Trypticase Soy Agar with 5% sheep blood (TSA), Columbia Agar with 5% sheep blood (CBA), MacConkey Agar (MAC) or Chocolate Agar (Chocolate). Chocolate was used for the cultivation of *Haemophilus* species only. All bacterial isolates were incubated aerobically for 18–24 hours at 35±2°C and were then tested on the MBT-CA System within 12 hours after incubation. Gram staining was performed on each isolate to confirm that it was gram-negative bacteria before MBT-CA testing. Frozen specimens were subcultured twice on appropriate plate media before testing was performed. A total of 2,263 specimens were collected at 5 separate study sites with site-specific institutional review board (IRB)-approved protocols. Specimens were then tested on the MBT-CA System and sent for sequencing (Accugenix, Newark, DE).

### Preparation of isolates for MBT-CA analysis—"Direct Transfer (DT)"

An individual colony from an overnight subculture plate was transferred to a selected position on an US IVD 48 Spot Target (target) using a sterile colony transfer device and reconstituted US IVD HCCA portioned (matrix) solution (1 μl) was added. The standard solvent (50% acetonitrile / 47.5% H_2_O / 2.5% trifluoroacetic acid) in the matrix solution extracts proteins (primarily ribosomal proteins) from the microorganisms. For validation, two US IVD bacterial test standard (BTS) control positions were selected on the target to inoculate with reconstituted BTS solution (1 μl, each). After drying at room temperature, BTS spots were overlaid with reconstituted matrix solution (1 μl, each). The inoculated target was dried and ready to be analyzed on the MBT-CA System.

### Preparation of isolates for MBT-CA analysis—"Extraction (Ext)"

If initial analysis with the MBT-CA did not produce a bacterial identification with a log(score) value of ≥ 2.0, or yielded a result of “not identified”, the organism was processed using the extraction procedure described in the package insert and the analysis was repeated. Briefly, a sterile 1 μl inoculation loop was used to transfer isolated colonies into 300 μl of HPLC-grade water and mixed thoroughly. Using a pipette, 900 μl of pure ethanol was added to the suspension. After thoroughly mixing, the specimen was centrifuged for two minutes at 13,000–15,000 rpm. The supernatant was removed and the centrifugation step was repeated. Residual ethanol was removed by pipetting. After allowing the pellet to dry at room temperature for a minimum of 5 minutes, 25 μl of 70% aqueous formic acid was added to re-suspend the pellet, followed by 25 μl of acetonitrile, which was mixed by pipetting. The specimen was then centrifuged for two minutes at 13,000–15,000 rpm and 1 μl of supernatant was loaded onto the target plate. For validation, two BTS control positions were selected on the target to inoculate with reconstituted BTS solution (1 μl, each). The sample and BTS spots were dried at room temperature and overlaid with reconstituted matrix solution (1 μl, each). The inoculated target was dried and subsequently analyzed using the MBT-CA System.

### MALDI-TOF MS measurement

Isolates were analyzed using the MALDI-TOF MS instrument. The matrix absorbs UV light and transfers protons onto the extracted proteins. A laser in the MALDI-TOF MS irradiates the matrix sample composite, causing evaporation and release of positively charged intact proteins and peptides ("soft" ionization technique). These ions are electrostatically accelerated over a short distance and arrive in the flight tube at a mass-dependent speed. As different proteins/peptides have different masses, ions arrive at the detector at different times (time-of-flight). The system measures the time (in the nanosecond range) between pulsed acceleration and the corresponding detector signal, the time is converted into an exact molecular mass.

Highly abundant microbial proteins (mainly ribosomal proteins) result in a mass spectrum with characteristic mass and intensity distribution. The distribution of mass and intensity creates a composite profile that acts as a molecular fingerprint to identify the test organism. A composite profile (mass spectrum) of proteins with *m/z* ratios of 2,000 to 20,000 was generated automatically by the system. Data acquisition as well as instrument quality control (e.g. calibration) was controlled by the MBT-CA Software.

### MBT-CA identification

The spectrum of the test organism was first transformed into a peak list. Using a biostatistical algorithm, this peak list was compared to the reference peak lists of organisms in the reference library (database) and a confidence score [log(score)] was generated. The mass spectrum from the test organism was then compared against each single entry of the MBT-CA reference library. In the MBT-CA library a species is usually represented by 5 to 10 individual strain entries from the same species. A higher log(score) indicates a higher degree of similarity to the organism in the reference library. Species identification is reported with "high confidence" if the log(score) is ≥2.00. A species identification is reported with "low confidence" if the log(score) is between 1.70 and <2.00. Any results <1.70 were considered as an unacceptable identification. Isolates that had a log(score) result <2.00 were re-tested following the extraction protocol described above.

### Strain collection—Reference laboratory

A TSA or Chocolate Agar slant from each isolate was subcultured at the study sites, incubated for 18–24 hours at 35±2°C, and sent to the reference laboratory (Medical College of Wisconsin, Milwaukee, WI). Upon arrival, isolates were subcultured to TSA plate and incubated for 18–24 hours at 35±2°C. A 3.0–4.0 McFarland dilution in saline was prepared for each specimen and used for nucleic acid extraction (QIAsymhony Pathogen extraction kit) as well as for cryo stock preparation following the package insert. Extracted DNA was frozen at -20°C and shipped to sequencing provider (Accugenix, 223 Lake Drive, Newark, DE) for bidirectional sequencing.

### Species delineation, "Reference Algorithm"—statistical analysis

Identification of isolates was initially performed using 16S rRNA gene sequencing according to CLSI MM18-A rules [14]. If application of MM18 rules was not possible (e.g. underrun thresholds, more than one species over thresholds) biochemistry and/or sequencing of further genes were used to verify the species delineation. Biochemical testing was performed by running isolates on the Vitek 2 (BioMérieux, France). If the isolate failed to show sufficient taxonomical resolution targeted protein sequence was used for further identification. A list of genes used for identification can be found in S1 Table.

Performance characteristics, including sensitivity and specificity, were calculated using standard methods. Ninety-five-percent confidence intervals were calculated by using a binomial expansion.

### Ethics Statement

Institutional review board (IRB) approval was obtained for this study by all sites that performed testing. These IRBs used for this study were the Medical College of Wisconsin and Froedtert Hospital institutional review board (Medical College of Wisconsin), Pearl (Laboratory Alliance), Institutional Review Board Cleveland Clinic Foundation (Cleveland Clinic), Kaiser Permanente Southern California Institutional Review Board (Kaiser Permanente) and University of New Mexico Health Sciences Center Human Research Review Committee (TriCore). This study, in accordance with the April 25, 2006 FDA guidance, was exempt from requiring informed consent because all specimens used in this study were residual clinical specimens that were de-identified prior to transfer to study staff. De-identification was performed in accordance to local IRB guidelines and each site used an “honest broker” who removed any linking identifiers prior to giving specimens to study staff to minimize patient health information risk. No specimens were collected specifically for the purpose of the study.

## Results

A total of 2,263 gram-negative, aerobic bacteria were isolated from 5 laboratory sites representing different geographical areas of the U.S. Isolates were analyzed using multi parameter analysis determined by each site’s standard of practice (bi-directional 16S rRNA gene sequencing, biochemistry, gene sequencing) compared to the MALDI Biotyper CA System. MBT-CA analysis identified 23 different genera consisting of at least 61 different bacterial species. Of the 2,263 isolates, 99.9% (2,261/2,263) produced acceptable identification log(scores) of ≥1.70. High confidence species identifications [log(score) ≥2.0] were generated for 96.8% (2,190/2,263) of the isolates and 3.1% (71/2,263) of the isolates were reported with low confidence [log(score) ≥1.70–<2.00]. The remaining 2 isolates failed to report acceptable confidence scores and were reported as no identification by the MBT-CA System (Table 1). The two isolates for which “no identifications” were reported had confidence scores of 1.53 and 1.64; these were identified as P*seudomonas putida* and *Stenotrophomonas maltophilia* by the reference algorithm.

**Table 1:** Details of MTB-CA System no ID strains.

Reference Algorithm ID	MBT-CA results	log(score)
*Pseudomonas putida* group	No ID	1.530
*Stenotrophomonas maltophilia*	No ID	1.638

### Performance of MBT-CA System compared to "Reference Algorithm"

Across all 5 laboratory testing sites, the MTB-CA System correctly identified, to the species/complex level, 98.2% (2,222/2,263) of all isolates tested. These results were consistent across 4 of the 5 sites, where concordance ranged from 99.1–99.5% (Table 2). The laboratory site 5 had a lower concordance, as determined by 95% confidence intervals, with an overall concordance of 88.2% (CI 82.9–92.1%). This site tested 18 strains of *Haemophilus haemolyticus* to determine whether *H*. *haemolyticus* could be differentiated from *H*. *influenzae*. As these species could not be differentiated reliably by the MTB-CA, the performance of this site was decreased. Removing these 18 isolates from the calculation raises the concordance to 96.4%. Comparing both high and low confidence results to the reference method for the identification to genus level, the MBT-CA correctly identified 99.8% (2,258/2,263) of all organisms tested.

**Table 2:** Performance of MBT-CA System

Test Site	High Confidence positives	High Confidence negatives	Low Confidence positives	Low confidence negatives	Negative	Total	% High confidence positive	% Low positive correct	% No ID
Site 1	801	5	11	1	0	818	97.9	1.34	0.0
Site 2	432	2	9	2	0	445	97.1	2.02	0.0
Site 3	355	1	7	1	0	364	97.5	1.92	0.0
Site 4	403	1	17	1	2	424	95.05	4.01	0.47
Site 5	183	7	4	18	0	212	86.3	1.89	0
All Sites	2174	16	48	23	2	2263	96.07	2.12	0.09

The MBT-CA System correctly identified 47 of the 61 different species tested without any error when species identification was constrained to high confidence results. Including the specimens that reported low confidence for identification an additional 7 species were correctly identified by the system without error (no mis-identification or no identification results). A summary of these species/complexes can be found in Table 3. Of the 7 remaining species tested, 6 of the 7 correctly identified ≥ 95% of the tested isolates. *H*. *influenzae* was the only species tested that performed poorly with only 71% of the isolates correctly identified.

**Table 3:** Performance of MBT-CA System for identification of aerobic Gram negative rods.

		Correct Identification			No ID	Discordant	
Species	# of isolates	Species Confirmation high confidence	Species Confirmation low confidence	Combined performance		Species Confirmation high confidence	Species Confirmation low confidence
*Achromobacter xylosoxidans*	75	70 (93.3%)	4 (5.3%)	74 (98.7%)			1 (1.3%)
*Acinetobacter baumannii complex*	70	67 (95.7%)	3 (4.3%)	70 (100%)			
*Acinetobacter Iwoffii*	69	69 (100%)		69 (100%)			
*Acinetobacter radioresistens*	27	27 (100%)		27 (100%)			
*Acinetobacter ursingii*	50	49 (98%)	1 (2%)	50 (100%)			
*Aeromonas sp [7]*	56	56 (100%)		56 (100%)			
*Alcaligenes faecalis*	26	26 (100%)		26 (100%)			
*Burkholderia gladioli*	6	6 (100%)		6 (100%)			
*Burkholderia multivorans*	19	19 (100%)		19 (100%)			
*Burkholderia cepacia complex [13]*	29	29 (100%)		29 (100%)			
*Citrobacter amalonaticus complex*	64	62 (96.9%)		62 (96.9%)		2 (3.1%)	
*Citrobacter koseri*	89	89 (100%)		89 (100%)			
*Citrobacter freundii complex*	89	89 (100%)		89 (100%)			
*Eikenella corrodens*	16	16 (100%)		16 (100%)			
*Enterobacter aerogenes*	80	80 (100%)		80 (100%)			
*Enterobacter cloacae complex*	95	74 (77.9%)	16 (16.8%)	90 (94.7%)		5 (5.3%)	
*Escherichia coli*	122	122 (100%)		122 (100%)			
*Haemophilus influenzae*	95	63 (66.3%)	4 (4.2%)	67 (70.5%)		8 (8.4%)	20 (21.1%)
*Haemophilus parainfluenzae*	34	32 (94.1%)	2 (5.9%)	34 (100%)			
*Hafnia alvei*	45	45 (100%)		45 (100%)			
*Klebsiella pneumoniae*	101	101 (100%)		101 (100%)			
*Klebsiella oxytoca Raoultella ornithinolytica*	68	68 (100%)		68 (100%)			
*Moraxella sg Branhamella catarrhalis*	66	66 (100%)		66 (100%)			
*Moraxella sg Moraxella osloensis*	28	28 (100%)		28 (100%)			
*Morganella morganii*	80	80 (100%)		80 (100%)			
*Pantoea agglomerans*	27	27 (100%)		27 (100%)			
*Pasteurella multocida*	46	46 (100%)		46 (100%)			
*Proteus mirabilis*	67	67 (100%)		67 (100%)			
*Proteus vulgaris group*	48	48 (100%)		48 (100%)			
*Providencia rettgeri*	55	50 (90.9%)	5 (9.1%)	55 (100%)			
*Providencia stuartii*	56	54 (96.4%)		54 (96.4%)			2 (3.6%)
*Pseudomonas aeruginosa*	78	78 (100%)		78 (100%)			
*Pseudomonas fluorescens group*	19	19 (100%)		19 (100%)			
*Pseudomonas putida group*	61	47 (77%)	13 (21.3%)	60 (98.4%)	1 (1.6%)		
*Salmonella sp*	86	86 (100%)		86 (100%)			
*Serratia liquefaciens*	28	28 (100%)		28 (100%)			
*Serratia marcescens*	69	69 (100%)		69 (100%)			
*Strenotrophomonas maltophilia*	76	75 (98.7%)		75 (98.7%)	1 (1.3%)		
*Yersinia enterocolitica*	44	43 (97.7%)		43 (97.7%)		1 (2.3%)	
*Yersinia pseudotuberculosis*	4	4 (100%)		4 (100%)			
All Isolates	2263	2174 (96.1%)	48 (2.1%)	2222 (98.2%)	2 (0.1%)	16 (0.7%)	23 (1%)

### Analysis of discordant results

A total of 1.8% (39/2,263) of the isolates were observed to be discordant to sequence analysis. Sixteen discordant isolates reported high confidence and 23 discordant isolates reported low confidence. Results for each non-*Haemophilus* discordant isolates with the resulting sequence ID are presented in Table 4. Discordant results that reported as high confidence were: 5 *Enterobacter amnigenus* which were reported as *Enterobacter cloacae* complex by the MBT-CA System, 8 *H*. *haemolyticus* were reported as *H*. *influenzae*, and 1 *Yersinia aldovae* was reported as *Yersinia enterocolitica*. Two isolates that were reported as *Citrobacter amalonaticus* complex (high confidence) by the MBT-CA System could not be confirmed by Reference Algorithm. Discordant results reported with low confidence contained 2 *Providencia rettgeri* reported as *Providencia stuartii*, 1 *Bordetella bronchiseptica* reported as *Acromobacter xylosoxidans*, and the remaining 20 discordant results were identified as *H*. *haemolyticus* by sequence analysis, but were reported as *H*. *influenzae* by the MBT-CA System.

**Table 4:** Details of incorrect identifications.

Species / Group/ Complex	MBT-CA result	log(score)	Reference method identification
*Achromobacter xylosoxidans*	*A*. *xylosoxidans*	1.810	*Bordetella bronchiseptica*
*C*. *amalonaticus* complex	*C*. *amalonaticus* complex	2.584	Undetermined^a^
*C*. *amalonaticus* complex	*C*. *amalonaticus* complex	2.513	Undetermined^a^
*E*. *cloacae* complex	*E*. *cloacae* complex	2.240	*Enterobacter amnigenus*
*E*. *cloacae* complex	*E*. *cloacae* complex	2.461	*Enterobacter amnigenus*
*E*. *cloacae* complex	*E*. *cloacae* complex	2.490	*Enterobacter amnigenus*
*E*. *cloacae* complex	*E*. *cloacae* complex	2.447	*Enterobacter amnigenus*
*E*. *cloacae* complex	*E*. *cloacae* complex	2.415	*Enterobacter amnigenus*
*Providencia stuartii / rettgeri*	*P*. *stuartii*	1.806	*Providencia rettgeri*
*Providencia stuartii / rettgeri*	*P*. *stuartii*	1.899	*Providencia rettgeri*
*Yersinia enterocolitica*	*Yersinia enterocolitica*	2.043	*Yersinia aldovae* ^b^

^a^ The reference method did not confirm any species

^b^ *Yersina aldovae* was fprmerly reported as a member of the *Y*. *enterolitica* like group X2

### Enhanced performance of MBT-CA System with further Extraction method

Any bacterial identification with a log(score) <2.00 was repeated following an extraction procedure. A total of 6.8% (153/2,263, including discordant results) of the enrolled isolates were re-tested using an extraction preparation (Table 5). Extraction resulted in an average increase of confidence score by 0.36.

**Table 5:** Performance of extraction method MBT-CA for identification of Gram negative bacteria.

Specimen ID Direct Transfer	Specimen ID after Extraction	Number of Isolates	Percent of Isolates
High Confidence	N/A*	2094	92.2
Low Confidence	High Confidence	64	2.88
Low Confidence	Low Confidence	34	1.53
Low Confidence	No ID	4	0.18
No ID	High Confidence	16	0.72
No ID	Low Confidence	10	0.45
No ID	No ID	2	0.09

*—extraction procedure not needed.

One hundred and two isolates that were extracted initially reported (direct transfer) as a low confidence score (≥1.7-<2.0). Extractions of these isolates increased the identification score from low to high confidence in 63% (64/102) of isolates tested, resulted in no change category (remain low confidence) confidence score in 33% (34/102) of isolates and resulted in a decrease from low to no ID in 4.0% (4/102) isolates tested. All isolates that resulted in a decrease to no ID were *Enterobacter cloacae* complex by sequence analysis. On average, extractions of low confidence score isolates raised the confidence score by 0.14.

Twenty eight isolates that were extracted initially were reported as no ID. Extractions increased scores to a reportable identification in 92.8% (26/28) of the no ID isolates. Direct transfer preparations initially reported as no ID were reported as high confidence in 57% (16/28) of the isolates and raised from no ID to low confidence in 36% (10/28) of the isolates. On average, extraction of no ID scores raised confidence score by 1.24. Ten isolates that were reported as no ID from whole cell preparation were initially reported with a confidence score of 0.00 (no mass spectrum acquired). These 10 isolates skewed the average increase from extraction process due to the low number of no ID isolates and the large increase in confidence score (average 2.28). When these 10 isolates were removed from the change-in-confidence score calculations, extraction of no ID isolates compared to direct transfer preparation increased on average by 0.44. The average of all extracted isolates (removing the no mass spectrum isolates) raised the score by 0.18 compared to direct transfer.

## Discussion

An abundant collection of literature demonstrates numerous benefits for the use of MALDI-TOF MS for microorganism identification [15–20]. In one study, the authors found the technology to more accurately identify rare bacteria than conventional phenotypic identification [21]. Furthermore when compared with traditional methods MALDI-TOF MS significantly reduced turnaround time [1, 21–23] and was more cost effective [1, 24]. On average, MALDI identifications reduced TATs by 1.45 days compared to current clinical protocols and reduce total identification costs by 56.9% [1]. These characteristics make incorporation of MALDI-TOF MS into the clinical laboratory an attractive option. The data presented here using a FDA-cleared system is consistent with these other findings from Research Use Only instruments demonstrating that MALDI-TOF MS is an accurate system for bacterial identification. In addition the system was equivalent across multiple sites demonstrating that laboratory to laboratory variation should not affect performance.

Laboratories who switch to using this instrument for all laboratory testing should be aware of some of the limitations of the system. With the current library the MBT-CA System was unable to differentiate between *H*. *influenzae* and *H*. *haemolyticus*. Differentiation is clinically important as *H*. *influenzae* can cause meningitis, epiglottitis, orbital cellulitis, and bacteremia [25], whereas, *H*. *haemolyticus*, is a commensal organism of the oropharynx and is generally considered nonpathogenic [26]. Differentiation of these two strains can be difficult due to similarities in biochemicals and so a quick and accurate method for differentiation would be helpful for patient management. Although the library used in the study cannot differentiate either species, Zhu *et al*. developed an additional reference library that added 20 strains (10 *H*. *influenzae* and 10 *H*. *haemolyticus*) [27]. The incorporation of these additional references into the MBT-RUO database allowed for the differentiation of *H*. *influenzae from H*. *haemolyticus* (n = 52). A similar study comparing 277 *Haemophilus* isolates demonstrated that the addition of a custom library increased the identification agreement to 99.6% as compared to multilocus sequence typing [28]. Both studies demonstrated the ability of the MBT-CA System to improve *Haemophilus* species identification. These libraries will likely be added by the manufacture, but until the new library is FDA-cleared laboratories wanting to use MALDI-TOF MS for differentiation of *haemophilus* will have to perform an in house laboratory validation.

Our study also identified a few additional mis-identifications compared to reference testing; however all but one of these were match the reference testing at the genus level. The one genus level discordant result identified was an isolate of *B*. *bronchiseptica* that was identified as *A*. *xylosoxidans* with low confidence. These two genera are closely related and belong to the same taxonomic family (*Alcaligenaceae*). Differentiation between *Brodetella* and *Acromobacter* has been previously shown to be difficult [29, 30]. As this was the only *Bordetella* isolate tested in this study it is unknown how often the system would generate mis-identification. Laboratories concerned for this can look at the source, patient symptoms and standard microbiological techniques for confirmation. The remaining mis-identifications were correctly identified to the genus level and would have had little clinical significance for patient care. Two isolates, a *P*. *putida* group member and a *S*. *maltophilia* isolate, failed to produce an acceptable identification and would require identification through alternative means.

Optimization of specimen preparation for MALDI-TOF MS identification has often included an extraction method to increase ribosomal protein yield and produce better spectra [31–33]. In this study any specimen that did not receive a high confidence score was treated to an extraction prior to testing to determine if the spectra could be increased to a high confidence score. In general our data is consistent with the literature that extraction of the specimen prior to testing increases the quality of spectra generation. However, performing an extraction is labor intensive and the data from this study demonstrated that extractions is unnecessary for most isolates (93.2% isolates tested). Extraction did increase yield better scores for the majority of specimens tested. A likely workflow for laboratories using this system would be to perform direct testing initially and then reflex isolates for extraction when scores result below 2.0.

A limitation to this study was that all testing was performed from bacteria grown at a uniform temperature, for a defined period of time using a limited number of growth media [34, 35]. All isolates were subcultured to TSA, CBA, MAC or Chocolate and incubated overnight at 35±2°C. Any clinical isolate that are grown on any additional agars or not incubated for 18–24h at 35±2°C would need to be considered separately. It is well known that growth on specific agar can affect MALDI-TOF MS protein profiles, resulting in incorrect identifications. One study evaluated the performance of the Bruker Biotyper for the identification of gram-negative bacteria using different growth media, namely Hektoen enteric medium and MacConkey agar [13]. This study demonstrated that some media reduced the rate of successful identification (log(score ≥2.0)), however, the growth medium did not lead increased mis-identifications. These data suggest that the MBT-CA System could uses different media without increasing misidentifications, but laboratories will need to perform verification studies.

To date, there are two FDA-cleared MALDI-TOF MS systems commercially available (Bruker MBT-CA System and Vitek MS [bioMerieux, Durham, NC). Several studies have reported high accuracy for identification that is equivalent to biochemical testing compared to both MALDI-TOF MS systems. Identifications with either system can be performed within minutes, at a substantial savings in cost per identification with an accuracy approaching 95% [10, 24, 36]. There have been some minor differences observed between the two systems. In one study the Bruker MBT-RUO system was found to be more reliable at a combined species/complex/genus level of identification than the Vitek MS RUO system (97.0% versus 89.5% P = 0.004), but the MBT-CA system required isolate extraction more often than the Vitek MS RUO [37]. However, as MALDI-TOF MS databases are always expanding there is limited utility in comparing current literature with older studies. Overall, the literature demonstrates that the MBT-CA System and the Vitek MS are comparable in their ability to correctly identify aerobic gram-negative bacteria [38–41].

In conclusion, we described the use of the Bruker MBT-CA System as a reliable method for identifying aerobic gram-negative bacteria recovered on solid media. In this study we found that the performance of the MBT-CA showed 98.2% correct identifications on the species/complex level and 99.8% on the genus level. Additional studies are necessary to establish FDA clearance of the MBT-CA System for other organisms such as gram positive bacteria, anaerobic bacteria, and yeast. The FDA clearance for aerobic gram negative bacteria does facilitate the incorporation of this technology into the clinical laboratory, which will promote the development of an automated MALDI-TOF MS system for routine bacterial identification.

## Supporting Information

S1 Table Gene sequenced for reference identification of isolates. (DOCX)
