# Supplementary material for: Multicenter Evaluation of the Bruker MALDI Biotyper CA System for the Identification of Clinical Aerobic Gram-Negative Bacterial Isolates
Source: PLoS One. 2015 Nov 3;10(11):e0141350. doi: 10.1371/journal.pone.0141350 (PMC4631355; doi:10.1371/journal.pone.0141350)
Supplement: S1 Table — (DOCX) [file pone.0141350.s001.docx]

| Supplemental Table 1. Gene sequenced for reference identification of isolates | |
| --- | --- |
| Organism | Gene |
| Achromobacter xylosoxidans | *recA* |
| Aeromonas sp[7] | *gyrB* |
| Burkholderia_cepacia complex_[13] | *recA* |
| Burkholderia_multivorans | *recA* |
| Citrobacter amalonaticus_complex | *tuf* |
| Citrobacter koseri | *tuf* |
| Citrobacter_freundii complex_[1] | *tuf* |
| Cronobacter dublinensis | *tuf* |
| Cronobacter sakazakii | *tuf* |
| Cronobacter turicensis | *tuf* |
| Delftia acidovorans | *gyrB* |
| Enterobacter aerogenes | *tuf* |
| Enterobacter amnigenus | *tuf* |
| Enterobacter asburiae | *tuf* |
| Enterobacter cloacae_complex | *tuf* |
| Escherichia_coli Shigella_sp | *tuf* |
| Haemophilus haemolyticus | *rpoB* |
| Haemophilus influenzae | *gyrB* |
| Haemophilus influenzae | *rpoB* |
| Hafnia alvei | *tuf* |
| Klebsiella pneumonia | *tuf* |
| Klebsiella_oxytoca Raoultella_ornithinolytica | *tuf* |
| Moraxella_sg_Moraxella osloensis | *recA* |
| Pantoea agglomerans | *gyrB* |
| Providencia rettgeri | *rpoB* |
| Pseudomonas aeruginosa | *gyrB* |
| Pseudomonas fluorescens_group | *gyrB* |
| Pseudomonas putida_group | *gyrB* |
| Pseudomonas putida_group | *tuf* |
| Serratia liquefaciens | *tuf* |
| Serratia marcescens | *tuf* |
| Serratia plymuthica | *tuf* |
| Serratia proteamaculans | *tuf* |
| Yersinia pseudotuberculosis | *tuf* |

S1 Table: The reference algorithm included specific gene sequencing for organisms were 16S ribosomal sequence analysis and biochemical methods were not efficient. These gene targets include *tuf, recA, gyrB* and *rpoB*.
